# Adult‐onset rapidly worsening progressive myoclonic epilepsy caused by a novel variant in DHDDS

**DOI:** 10.1002/acn3.51483

**Published:** 2021-11-27

**Authors:** Seondeuk Kim, Man Jin Kim, Hyoshin Son, Sungeun Hwang, Mi‐Kyoung Kang, Kon Chu, Sang Kun Lee, Jangsup Moon

**Affiliations:** ^1^ Department of Neurology Seoul National University Hospital Seoul South Korea; ^2^ Laboratory for Neurotherapeutics Seoul National University Hospital Seoul National University College of Medicine Seoul South Korea; ^3^ Rare Disease Center Department of Genomic Medicine Seoul National University Hospital Seoul South Korea

## Abstract

Progressive myoclonic epilepsy (PME) is a heterogeneous neurogenetic disorder manifesting as progressive myoclonus, seizure, and ataxia. We report a case of PME caused by a novel *DHDDS* variant. Additionally, by reviewing the literature on *DHDDS* mutations, we compared the phenotype of our patient with previously reported phenotypes. We identified *DHDDS (c.638G>A, p*. *Ser213Asn)* as a likely pathogenic variant. The literature review revealed 15 PME patients with *DHDDS* mutations from 13 unrelated families. According to previous studies, late‐onset patients tend to have a slow‐progressive disease course. Although his myoclonus and ataxia were adult onset, our patient experienced rapid disease aggravation.

## Introduction

Progressive myoclonic epilepsy (PME) manifests as progressive and disabling myoclonus, generalized or focal seizure, and ataxia. However, patients with PME show clinical and genetic heterogeneity.^1^ Recently, novel genetic mutations associated with PME were discovered by panel sequencing or whole‐exome sequencing (WES).^2^ Among the causative genetic mutations, attention was paid to the genes related to the dolichol‐dependent protein glycosylation pathway. *NUS1*, *DHDDS*, and *ALG10* were identified as the genes with pathogenic variants inducing the manifestation of PME.^3^ In this article, we describe a PME patient with a novel *DHDDS* variant.

## Methods

### Genetic analyses

Using the SureSelectXT Human all Exon 60 Mb Kit (Agilent Technologies, Santa Clara, CA) and the HiSeq 2500 sequencing system (Illumina, San Diego, CA), we performed WES on the proband’s blood sample. The DNA sequence was aligned to the reference genome (UCSC hg19) and analyzed. The variant’s pathogenicity was classified according to the standards and guidelines of the American College of Medical Genetics and Genomics (ACMG).^4^ We used the Genome Aggregation Database (http://gnomad.broadinstitute.org/) to assess pathogenicity. Among the in silico programs, SIFT, PolyPhen‐2, and MutationTaster were applied.^5^, ^6^, ^7^ For the verification of de novo mutations, both parental samples were tested for the variant by Sanger sequencing. When paternity or maternity could not be determined (e.g., deceased father or mother), we tested the other first‐degree relatives of the proband.

### Literature review

To date, congenital disorders of glycosylation, retinitis pigmentosa, and PME have been reported as phenotypes of *DHDDS* mutations. The first case of PME associated with *DHDDS* was reported in 2017.^2^ Therefore, we used PubMed and Google Scholar and searched recent publications (from January 2017 to July 2021) using the terms “epilepsy” and “DHDDS.”

## Results

### Case description

**Figure 1:**
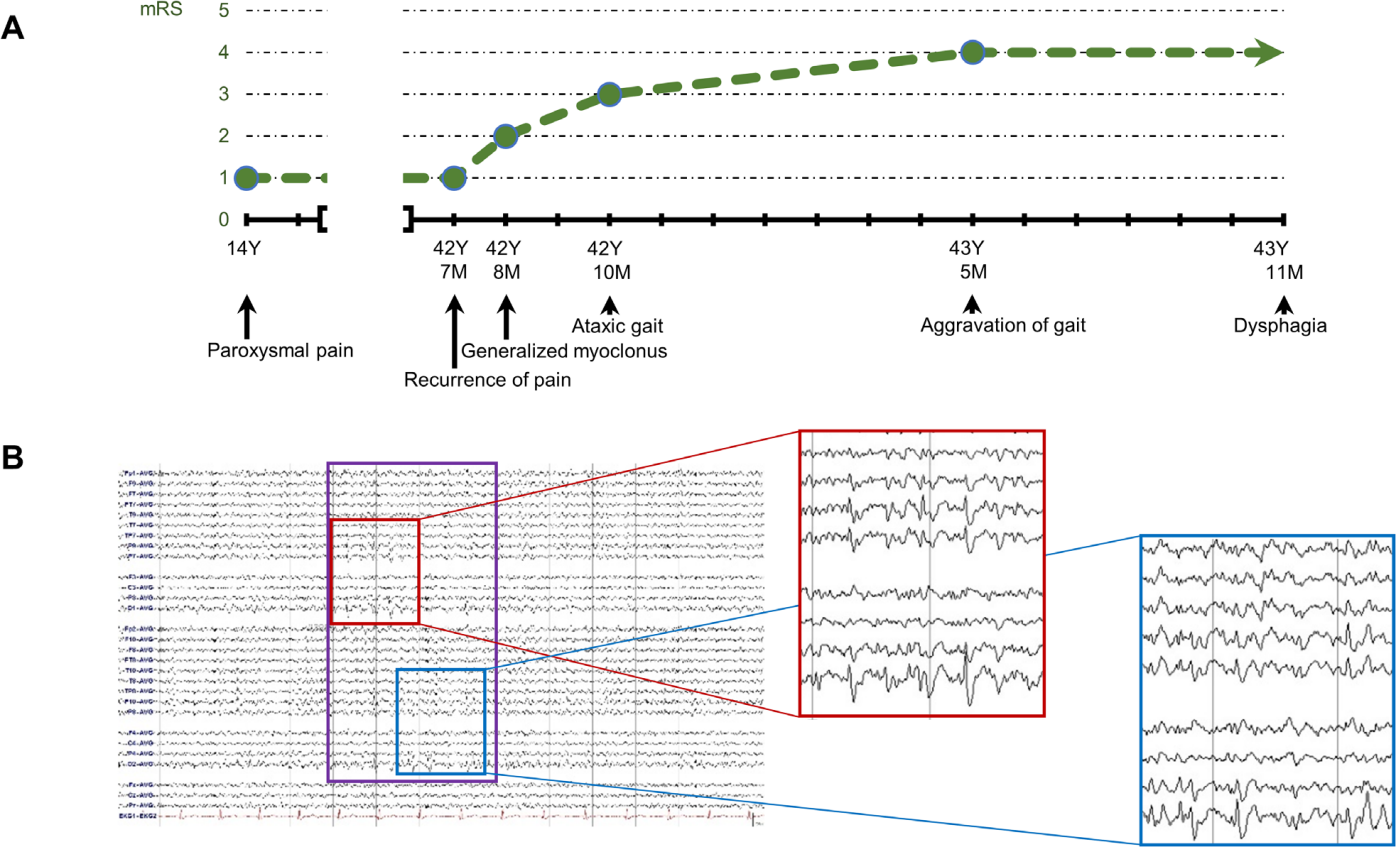
Clinical course and Electroencephalography (EEG). (A) The disease course of the patient. Since generalized myoclonus appeared, he showed rapid progression of myoclonus and ataxia. (B) Average montage EEG of the patient. Within the purple rectangle, there are several right or left occipital spikes. Red rectangle highlights left occipital spikes. Blue rectangle highlights right occipital spikes.

A 42‐year‐old male patient visited our center complaining of progressive involuntary movement for 2 months. He had neither a history of a perinatal problem nor a family history of epilepsy or other neurological disorders. He had experienced normal motor and social development. Although he had graduated high school, his academic performance was at the bottom level. At 14 years of age, he presented with episodic aphasia. He was diagnosed with epilepsy and prescribed an antiepileptic drug (carbamazepine). Since then, the symptom was controlled. Approximately 3 months prior to the visit, there was one event of behavior and speech arrest with awareness. Two months ago, involuntary movement of the entire body, including eyelid myoclonus and tremulous head movements, has emerged. The movements were provoked by action and gradually deteriorated (Figure 1A).

On neurological examination, generalized myoclonus was provoked by action but persisted at resting status. He showed an ataxic gait characterized by a wide base, short step length, and irregular stride. Until then, he could walk without assistance. He demonstrated dysarthria but no dysphagia. The deep tendon reflex was increased, and the Hoffman sign was present on both sides. The Bedside MMSE score was 21 (time ‐1, calculation ‐5, comprehension ‐1, pentagon ‐1).

On 24 hours of video scalp EEG monitoring, bilateral occipital lobe spikes (Figure 1B) and generalized intermittent rhythmic delta slow activities were detected. His brain and whole‐spine MRI were unremarkable. In the skin biopsy, there was no Lafora body. Targeted sequencing for MERFF and DRPLA detected no mutations.

Seven months after the visit, he presented with aggravation of gait disturbance. He could not walk without assistance. After 1 year, he had difficulty in swallowing. At that time, however, follow‐up brain MRI revealed no significant interval change compared with the initial image (interval of 11 months).

### Identification of a de novo *DHDDS* mutation

**Figure 2:**
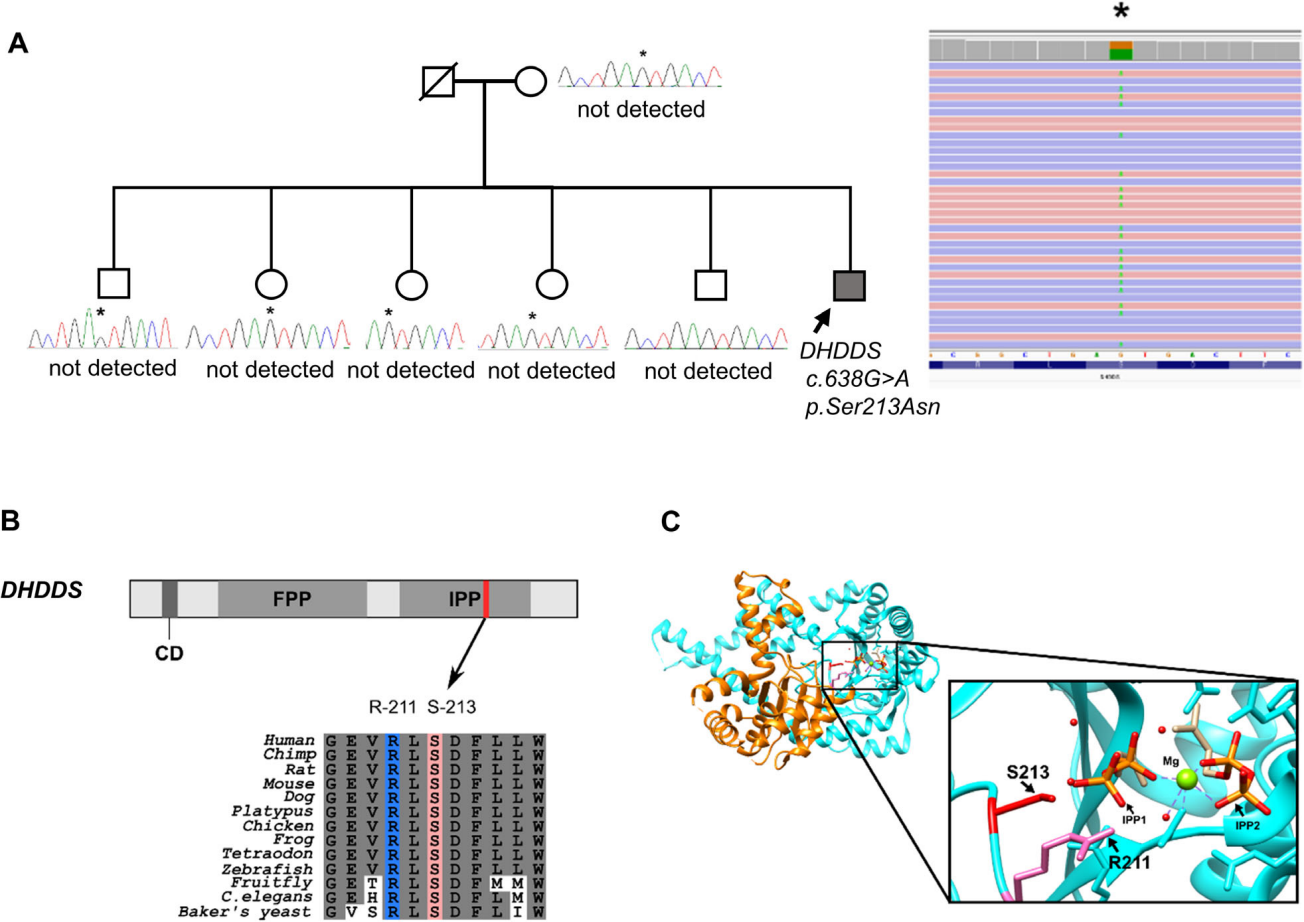
*DHDDS* (c.638G>A, p. Ser213Asn), heterozygote variant. (A) Pedigree of the family. *DHDDS*, p. Ser213Asn was not detected in the patient's mother or all of the siblings (two brothers and three sisters) (denoted with asterisks). (B) *DHDDS*, p. Ser213Asn is located in the isopentenyl diphosphate binding site domain (IPP), which is evolutionarily conserved across vertebrate, insect, and yeast genomes. The *DHDDS* Ser‐213 residue as well as a conserved arginine residue (Arg‐211) plays a critical role in binding between isopentenyl diphosphate and a DHDDS protein. (C) The overall structure and active site of NgBR/DHDDS complex. NgBR is colored in orange, and DHDDS is colored in cyan. Two IPP molecules and Mg2+ ion bound at the active site are shown. The pyrophosphate of IPP1 is tightly bound by DHDDS Arg‐211 and Ser‐213. The three‐dimensional structure image of NgBR/DHDDS complex (Protein Data Bank ID code 6W2L) was generated using the UCSF Chimera 1.14. CD, catalytic domain; IPP, isopentenyl diphosphate binding site; FPP, farnesyl diphosphate binding site; Mg, magnesium; ID, identification.

We identified *DHDDS* (c.638G>A, p. Ser213Asn)*, heterozygote*. The variant was absent in the population database. In multiple in silico programs, the variant was predicted as a pathogenic variant. The parent test was unavailable because the patient’s father had passed away. Therefore, blood samples were obtained from his mother and all of the siblings (two brothers and three sisters) for testing the *DHDDS* variant. The *DHDDS*, p. Ser213Asn was not detected in the patient's mother or all of the siblings. (Figure 2A) The *DHDDS*, p. Ser213Asn is located in the isopentenyl diphosphate binding site domain (IPP), which is evolutionarily conserved across vertebrate genome.^8^ (Figure 2B) Based on these results, the c.638G>A variant was assumed to be a de novo mutation. According to ACMG standards and guidelines, this variant was classified as a likely pathogenic variant [2 PM (PM2, PM6) + 2 PP (PP3, PP4)].^4^

### Reported *DHDDS* variants

In the literature review, the patients with *DHDDS* gene mutations demonstrated progressive myoclonus, ataxia, and generalized or focal epilepsy. Fifteen patients with *DHDDS* missense variants associated with PME or progressive myoclonic ataxia (PMA) have been described.^2^, ^3^, ^9^, ^10^, ^11^, ^12^, ^13^ (Table 1) The total number of *DHDDS* variants is five. The most common mutation is *DHDDS* c.632G>A (n = 6), followed by c.614G>A (n = 4) and c.110G>A (n = 3). Only one patient was reported to have *DHDDS* c.283G>A and c.109C>T. Almost all patients were considered to have de novo mutations. In one family, the mutation of two patients (11 and 12) was inherited from their mother (10).

**Table 1:** Literature review of progressive myoclonic epilepsy (PME)‐associated *DHDDS* gene mutations.

Number	Sex	Gene	Inheritance	Ancestry	Onset	Movement disorder	Epilepsy	GDD and/or ID	Brain MRI	EEG	Ref.
Cluster 1											
1	F	*DHDDS c.110G>A (p. Arg37His) (het)*	*de novo*	Canadian	Early	Myoclonus Onset unknown (However, last follow‐up age: 5 years 1 month)	Onset 18 months Myoclonic absence, photosensitive GTCS/Febrile focal seizure Intractable seizures	GDD ID: severe	normal	GSW, PPR	2
2	M	*DHDDS c.110G>A (p. Arg37His) (het)*	*de novo*	Canadian	Early	Myoclonus, Ataxia, and Tremor Onset unknown (However, last follow‐up age: 5 years 6 months)	Onset 1 YO Absence/Atonic/Fever sensitive VPA effective	GDD ID: unknown	normal	GSW, diffuse slowing	2
3	F	*DHDDS c.110G>A* *(p. Arg37His) (het)*	*de novo*	Japanese	Early	Myoclonus, Tremor, and Dystonia Onset myoclonic tremor (infancy), intention tremor (3 YO), resting tremor (16 YO) Worsened by postural/voluntary task Nonprogressive nature	Onset 3 YO eye blinking seizure (at 3 YO)/GTCS (15 YO) VPA effective	no GDD ID: mild (4 YO) ‐ IQ 40 (16 YO)	normal	GSW, PPR	11
4	F	*DHDDS c.632G>A (p. Arg211Gln) (het)*	*de novo*	Canadian	Early	Myoclonus, Ataxia, and Tremor Onset unknown (However, last follow‐up age: 5 years 6 months)	Onset 4 YO Myoclonic absence/OXBZ effective	GDD ID: mild borderline IQ	normal, Chiari I malformation	epileptiform discharges	2
5	M	*DHDDS c.632G>A (p. Arg211Gln) (het)*	*de novo*	Canadian	Early	Myoclonus, Tremor, and Parkinsonism Onset 6–9 YO Facial myokymia, bradykinesia, hypomimia, rigidity, freezing, and impaired postural reactions	Onset 6–9 YO No seizures since the age of 9 years	GDD ID: severe	normal	GPSW	2
6	F	*DHDDS c.632G>A (p. Arg211Gln) (het)*	*de novo*	Canadian	Early	Myoclonus, Ataxia, Tremor, and Dystonia Onset 7 YO	No epilepsy	GDD ID: moderate to severe	normal	normal	2
7	M	*DHDDS c.632G>A (p. Arg211Gln) (het)*	*de novo*	Italian	Early	Myoclonus and Ataxia Onset 7 YO Slowly progressive nature Mild ataxia	Onset 9 YO Absence with eyelid myoclonia	GDD ID: moderate	not reported	not reported	3
8	F	*DHDDS c.632G>A (p. Arg211Glna) (het)*	*de novo*	Australia	Early	Myoclonus Onset unknown (However, last follow‐up age 5 years 10 months)	Onset 6 mo. Absence with eyelid myoclonia Myoclonic‐atonic seizure Absence/Eyelid myoclonia GTCS/NCSE	GDD ID: unknown	normal	GSW (3–4 Hx), GPSW	13
9	M	*DHDDS c.632G>A (p. Arg211Glna) (het)*	*de novo*	Italian	Early	Myoclonus and choreoathetosis Onset 15 months Hyperkinetic behavior (stereotyped hand movement)	Onset 14 months GTCS/Refractory	GDD ID: mild	normal	GSW	12
10	F	*DHDDS c.614G>A (p. Arg205Gln) (het)*	*Unknown*	UK	Early	Tremor Onset: early childhood Nonprogressive	Onset 14 YO History of febrile Sz/LTG effective	no GDD ID: mild	normal	not reported	9
11	F	*DHDDSc.614G>A (p. Arg205Gln) (het)*	*Inherited* *(*Daughter of #13*)*	UK	Early	Myoclonus, ataxia, and orofacial dyskinesia Onset 3 YO Worsened from 7 YO	Onset 11 months Absences with eyelid myoclonia Atonic/Myoclonic Photosensitive, fever sensitive VPA, LEV partially effective	GDD ID: severe	normal	GSW, background slowing	9
12	F	*DHDDS c.614G>A (p. Arg205Gln) (het)*	*Inherited* *(*Daughter of #13*)*	UK	Early	Myoclonus, tremor, orofacial dyskinesia, and ataxia Onset 5 YO	Onset 12 months Absences with eyelid myoclonia Atonic seizure/LEV partially effective	GDD ID: moderate	normal	GSW	9
Cluster 2											
13	F	*DHDDS c.614G>A (p. Arg205Gln) (het)*	*de novo*	Italian	Late‐	Myoclonus and Ataxia Onset ataxia (late infancy), myoclonus (29 YO) mild and nonprogressive	Onset 17 YO TCS (rare)	no GDD ID: mild	mild diffuse atrophy	not reported	3
14	F	*DHDDS c.283G>A (p. Asp95Asn) (het)*	*de novo*	Italian	Late	Myoclonus, Ataxia, and tremor Onset tremor (21 YO), myoclonus (35 YO), ataxia (37 YO) Slowly progressive	No epilepsy Single TCS (36 YO)	no GDD no ID	cerebellar atrophy.	not reported	3
15	F	*DHDDS c.109C>T (p. Arg37Cys) (het)*	*de novo*	Korean	Late	Myoclonus and Ataxia Onset myoclonus (early 20 s), ataxia (onset 30 s) Slowly progressive nature	No definite clinical seizure. However, considered as epilepsy due to EEG	no GDD ID: not complete the elementary curriculum IQ 40(46 YO)	normal	GSW (3 Hz)	10
16*	M	*DHDDS c.638G>A (p. Ser213Asn) (het)*	*de novo*	Korean	Late	Myoclonus and Ataxia Onset 42 YO Rapidly progressive	Onset 14 YO Speech and behavior arrest with impaired awareness	no GDD ID: mild MMSE 21 (44 YO)	normal	bilateral occipital lobe spike, diffuse slowing,	

Abbreviations: FIAS, Focal impaired awareness seizure; GDD, generalized developmental delay; GPSW, generalized polyspikes and waves; GSW, generalized spikes and waves; ID, Intelligent disability; LE, Lower extremities; PPR, photoparoxysmal response; Ref., Reference; TCS, tonic‐clonic seizure; UE, Upper extremities; YO, years‐old.

*Our patient.

## Discussion

By the application of gene sequencing panels or WES in the discovery of germline mutations in adult epilepsy patients, various rare gene mutations have been reported in patients with a suspicion of PME or PMA.^2^, ^3^ In this article, we report a case of PME induced by a novel variant of *DHDDS*. Furthermore, we compared the phenotype of our patient with those of previously described patients with *DHDDS* mutations.

Since the genetics of PME are very heterogeneous, the patterns of ethnicity were studied according to the mutated genes.^14^ In the case of the *DHDDS* variant including our patient (n = 16), the patients were usually seen in Europe (44%, 7/16) or North America (31%, 5/16). Some, including our case, were confirmed in East Asia (19%, 3/16). Furthermore, there was only one patient (6%, 1/16) in Australia.

The age of onset is also different according to the genes involved.^1^ Silvana Franceschetti et al. reported the onset of PME in patients with various gene mutations. In addition, they also suggested that patients with PME with undetermined genes could be grouped into 2 clusters by two‐step cluster analysis based on categorical variables (psychomotor delay preceding myoclonus onset, EEG findings, etc.). In cluster 1, the patients demonstrated earlier onset of both neurological symptoms and myoclonus. On the other hand, the patients in cluster 2 often experienced adult‐onset symptoms. Cluster 1 tended to have psychomotor delay and recurrent seizures.^15^ Likewise, patients with *DHDDS* mutations could be classified as having early‐onset or late‐onset movement symptoms. Late onset can be defined as adult onset (>19 years old). As a previous study reported, there was no moderate to severe intellectual disability in the late‐onset group (0%, 0/4). In contrast, half (50%, 6/12) of the early‐onset group had moderate to severe intellectual disability (mild 33%; unknown 17%). Betul Baykan et al. reported that patients with late‐onset Lafora disease tend to progress slowly.^16^ In *DHDDS*, all the previously reported late‐onset patients also exhibited slowly progressive or nonprogressive movement symptoms. Meanwhile, our patient complained of movement symptoms in late adulthood (42 years old) but showed a rapidly progressive course of the symptoms. After approximately 10 months, he had a moderately severe disability and was unable to ambulate without assistance.

Various patterns of seizure and EEG abnormalities appear in PME.^1^ The seizure patterns include generalized tonic‐clonic, absence, atonic, focal impaired awareness seizures, eye blinking, and visual seizures. On EEG, generalized spikes and waves and a diffuse slow background are usually detected. Some patients have photosensitivity or focal epileptiform discharge. Occipital or posterior epileptiform discharge is seen in patients with Lafora’s disease or classic late infantile neuronal ceroid lipofuscinoses (NCL).^1^ Almost all patients with *DHDDS* variants are accompanied by generalized epilepsy, showing generalized tonic‐clonic seizures or absence seizures with rhythmic generalized or bifrontal dominant spikes and waves. In contrast, our patient had focal epilepsy, exhibiting behavior and speech arrest with impaired awareness. Scalp EEG revealed bilateral occipital spikes but not generalized epileptiform discharge, mimicking Lafora’s disease. Namely, the EEG patterns of the *DHDDS* variant varied like overall PME patients.

*NUS1* and *DHDDS* are the genes encoding cis‐prenyltransferase (cisPTase), which plays a critical role in farnesyl diphosphate metabolism. Among the recently reported mutations of *DHDDS*, one mutation affects the catalytic domain (amino acid positions 34–38) (p. Arg37), and another mutation is located in the substrate (isopentenyl pyrophosphates; IPPs) binding site (p. Arg211). Interestingly, all patients with the *DHDDS* c.110G>A (p. Arg37His) or c.632G>A (p. Arg211Gln) variant had early‐onset PME or PMA. DHDDS Ser‐213 is located at the active site of NgBR/DHDDS complex. A fully active DHDDS combined with NgBR (Nogo‐B receptor) catalyzes the condensation of a pyrophosphate with isopentenyl pyrophosphates, resulting in the formation of polyprenol pyrophosphates. The pyrophosphate with one of two isopentenyl pyrophosphates is tightly bound by DHDDS Arg‐211 and Ser‐213 (Figure 2C).^8^ The three‐dimensional structure image of NgBR/DHDDS complex (Protein Data Bank ID code 6W2L) was generated using the UCSF Chimera 1.14. Michal Lisnyansky Bar‐El et al. suggested that these loci are close to RxG motif or substrate binding region. The mutations of these sites could directly perturb the binding of substrate, significantly reduce the enzyme activity, and induce relatively severe epileptic phenotypes with autosomal dominant inheritance. On the other hand, the mutations of other sites, like Lys42Glu, could indirectly affect substrate coordination and mildly decrease the catalytic activity. This finding might be why *DHDDS*‐related retinitis pigmentosa is only induced by the mutations in both alleles, autosomal recessive inheritance, and shows the milder phenotypes.^17^ Therefore, the mechanism of this disease is thought to be attributed to enzyme activity. However, this presumption cannot explain why heterogeneous patterns (early onset vs. late onset) appeared in the epilepsy phenotype. Moreover, it is limited because two patients (Patient 13, 14) had decreased enzyme activity, and two patients (Patient 5, 6) had normal glycosylation assays. For these findings, the gain‐of‐function mechanism is considered the main mechanism. Nonetheless, our patient’s disease course, which was late onset but rapidly progressive, could not be explained by only this mechanism. There may be additional mechanisms associated with disease expression and progression. Notably, most mutations were the substitution of other amino acids for Arg. DHDDS plays a critical role in protein N‐glycosylation, a metabolic process attaching glycan to Asn or Arg of protein. Consequently, we presume that Asn or Arg residue of DHDDS might be important, and the substitution of other amino acids for Arg could significantly alter the process. In this regard, we propose that replacing Ser with Asn could also affect enzyme function in our patient with *DHDDS* p. Ser213Asn.

## Conclusion

We report a case of PME with a novel *DHDDS* variant (c.638G>A, p. Ser213Asn). From the literature review, PME caused by a *DHDDS* variant can be dichotomized into early‐ or late‐onset movement symptoms. Furthermore, patients with late‐onset PME have a slowly progressive disease course. However, some patients may exhibit a more various spectrum of phenotypes, like our patient, who was classified as having late‐onset disease but showed rapid progression of myoclonus and ataxia. Consequently, further studies on phenotypes and genetic mutations are warranted.

## Author Contributions

Seondeuk Kim: Drafting/revision of the manuscript for content, including medical writing for content; major role in the acquisition of data; analysis or interpretation of data. Man Jin Kim: Revision of the manuscript for content, including medical writing for content; major role in the acquisition of data; analysis or interpretation of data. Hyoshin Son: Analysis or interpretation of data. Sung Eun Hwang: Analysis or interpretation of data. Mi‐Kyoung Kang: Analysis or interpretation of data. Kon Chu: Study concept or design. Sang Kun: Study concept or design. Jangsup Moon: Study concept or design.

## Conflict of Interest

The authors declare no conflict of interest.

## Ethical Statements

The authors confirm that they have read the Journal’s position on issues involved in ethical publication and affirm that this report is consistent with those guidelines.
